# Diagnostic accuracy of otitis media with and without a fictitious AI support among physicians in primary care and medical students

**DOI:** 10.1080/02813432.2025.2571936

**Published:** 2025-10-15

**Authors:** Malin Hedman, Vezira Kosuta, Manfred Lindmark, Josefin Sandström, Brenda Trinh, Pär-Daniel Sundvall, Karin Rystedt, Mimmi Werner, Fredrik Öhberg, Thorbjörn Lundberg

**Affiliations:** ^a^ Department of Public Health and Clinical Medicine, Umeå University, Umeå, Sweden; ^b^ General Practice/Family Medicine, School of Public Health and Community Medicine, Institute of Medicine, Sahlgrenska Academy, University of Gothenburg, Gothenburg, Sweden; ^c^ Research, Education, Development & Innovation, Primary Health Care, Region Västra Götaland, Sweden; ^d^ Department of Diagnostics and Intervention, Umeå University, Umeå, Sweden; ^e^ Department of Clinical Sciences, Otorhinolaryngology, Umeå University, Umeå, Sweden

**Keywords:** Artificial intelligence, diagnostic confidence, diagnostic accuracy, otitis media, technological impact

## Abstract

**Background:**

Otitis media (OM) in children is a common infection in primary care, contributing to a significant global health and economic burden. In high-income countries, diagnostic inaccuracy leads to over-diagnosis of acute OM (AOM) and over-prescribing of antibiotics, which may contribute to antibiotic resistance.

**Aim:**

To investigate the diagnostic accuracy and the influence of artificial intelligence (AI) in diagnosing OM among primary care physicians and medical students.

**Method:**

A diagnostic accuracy study in which primary care physicians and medical students diagnosed AOM, OM with effusion (OME), and normal eardrums using 21 high-quality digital images, both without and with a fictitious AI support. We estimated the technological impact of the fictitious AI support.

**Results:**

Overall diagnostic accuracy was 64% without, and 75% with AI support. The most experienced physicians reached 69% without, and 80% with AI; the least experienced 61% without, and 73% with AI; medical students reached 64% without, and 74% with AI. Accuracy for AOM was 77% without and 86% with AI, and for OME 46% without and 66% with AI. Mean diagnostic confidence increased significantly with AI support. The technological impact was 1.4. Automation bias was 1.2 overall, 0.9 for the most experienced and 1.2 for the least experienced physicians.

**Conclusion:**

We report modest diagnostic accuracy for OM among primary care physicians and medical students. The fictitious AI support system improved both accuracy and diagnostic confidence and reduced over-diagnosis. The most experienced physicians achieved the highest accuracy, the less experienced were more often misled by the fictitious AI.

## Introduction

Otitis media (OM) is globally one of the most common infections in children causing a substantial burden of disease and a considerable economic impact [1,2]. OM has the following subgroups: OM with effusion (OME), acute OM (AOM) and chronic suppurative OM (CSOM). CSOM is an important reason for disabling hearing loss in children and result from recurrent or inadequately treated AOM, more commonly seen in low-income countries [1]. AOM is a common reason for prescribing antibiotics [1,3]. However, in high-income countries there is a tendency to over-diagnose AOM, which contributes to the over-use of antibiotics in children [4,5], a practice closely linked to the growing problem of antibiotic resistance [6]. Despite the importance of correct diagnosis, OM remains challenging to diagnose accurately in clinical practice. The diagnostic uncertainty underscores the need for improved tools and strategies to support accurate, evidence-based diagnosis and reduce unnecessary antibiotic use.

Most cases of AOM are seen in primary care. Studies are limited but indicate that general practitioners (GP) lack the competence and confidence to adequately examine and assess the eardrum [7]. The few studies on diagnostic accuracy in primary care show an accuracy of 36% to 71% with OME being more challenging to diagnose [8–10]. The diagnosis of OM is taught in medical school, but studies indicate a lack of practical training in otoscopy, as well as low confidence and accuracy in performing it among medical students [11,12].

An accurate diagnosis of OM usually requires both a visual assessment of the eardrum and an evaluation of eardrum mobility, the latter being essential for identifying middle ear effusion. For visual assessment, in addition to traditional otoscopy, digital otoscopy can be used to capture still images or video. This method has been found to improve accuracy overall compared to traditional otoscopy for both medical students and GPs [13–15]. For mobility assessment, pneumatic otoscopy and tympanometry are techniques that have been proven to increase the diagnostic accuracy of OM [16,17]. Although the availability of equipment to assess mobility can be limited, it is not always utilized in primary care even when available [18,19].

The adoption of artificial intelligence (AI) in medicine—as well as in other areas of society—is attracting considerable attention. In Swedish primary care settings, the current use of AI is limited to administrative support and ECG interpretation. However, there is significant potential for future applications in areas such as patient monitoring, diagnostics, and prognostics. In the context of OM diagnostics, AI can be employed for image classification in combination with digital otoscopy. Several systems have been evaluated in experimental settings [20–22], although none of these studies have been conducted within a primary care environment. When compared to physicians, AI may achieve higher diagnostic accuracy. As an example, in a study from 2023, clinicians had an accuracy of 65% in diagnosing AOM, OME and normal eardrums, while the AI algorithm used reached an accuracy of 96% on the same images [21]. Before introducing AI systems in clinical practice, it becomes relevant to evaluate what influence the system can exert on human judgement and decisions [23]. Studies on the interaction between physicians and AI support systems are limited. One study on skin cancer diagnostics found that physicians often changed their diagnosis when exposed to the AI system, both guiding and misleading in their decisions [24]. However, AI’s effects on clinicians in their OM diagnostics have not yet been investigated.

Given the limited knowledge about diagnostic accuracy for OM in primary care, both with and without AI support, two objectives were defined. First, the diagnostic accuracy of OM among medical students and primary care practitioners was evaluated under conditions both with, and without fictitious AI assistance. Second, the influence of a fictitious AI support on clinicians’ diagnostic decisions was assessed.

## Methods

This cross-sectional diagnostic accuracy study was conducted among physicians in primary care and medical students in five regions in Sweden between September 2023 and June 2024. Participants assessed digital images of eardrums, with and without AI support, via a structured questionnaire.

The Swedish Ethical Review Authority determined that the study does not fall under the provisions of the Swedish Ethical Review Act and therefore did not require formal ethical approval. In its advisory opinion, the authority also stated that it had no ethical objections to the study (Ref. no. 2023-03878-01).

### Participants

**Figure 1.**
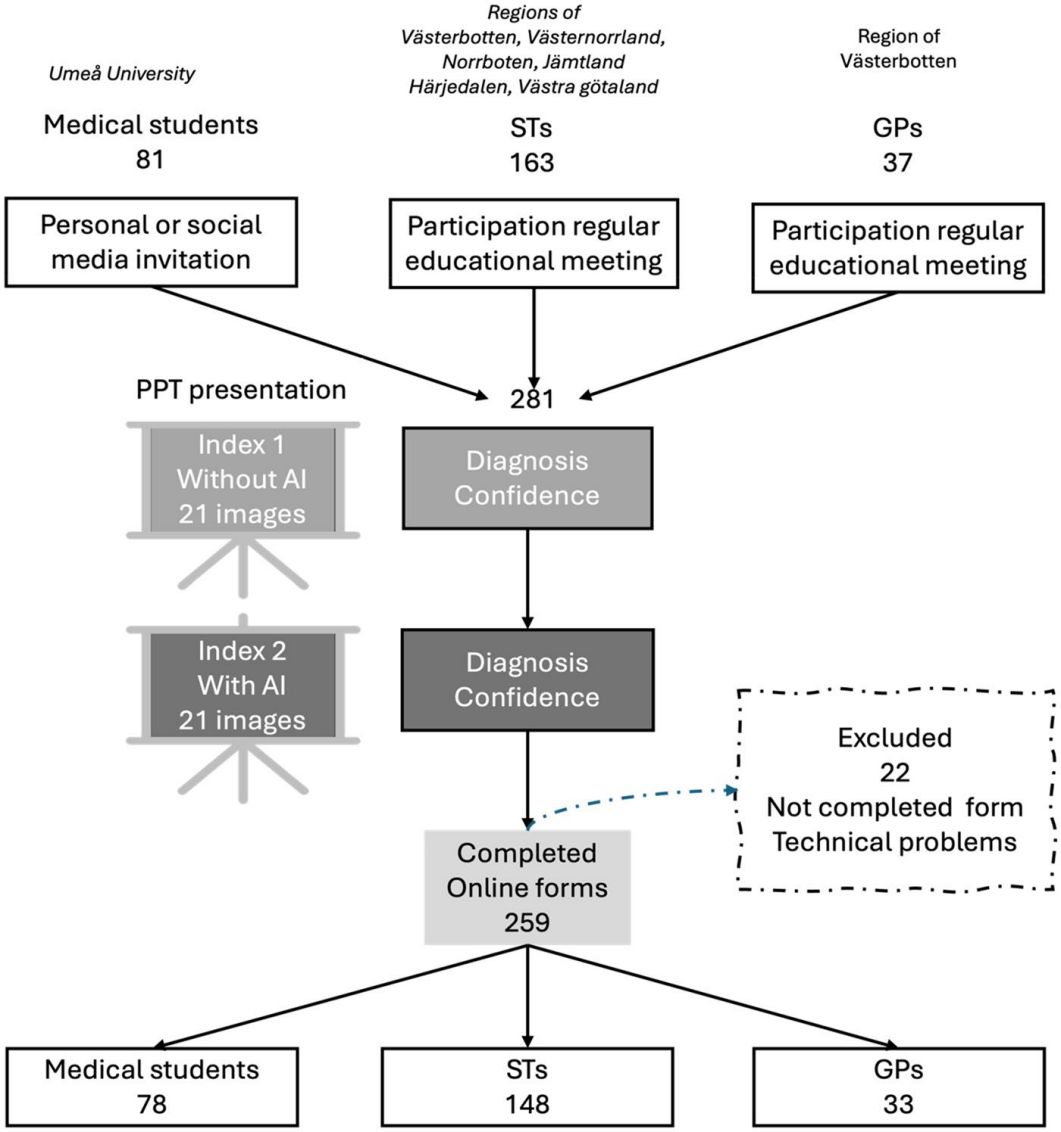
Flow chart of study. PowerPoint (PPT) presentations at meetings, registration of responses in an online form.

GP, specialist trainees in general practice (ST), and medical students who had completed their otolaryngology course were recruited from the regions of Västerbotten, Västernorrland, Jämtland/Härjedalen, Norrbotten and Västra Götaland (Figure 1). ST in general practice in Sweden is a five-year employment program including practical and theoretical education. Recruitment of GP and ST occurred during regular educational meetings, while medical students were invited *via* digital platforms targeting tenth-term students.

All participants received both verbal and written information about the study. Participation was anonymous, and no personally identifiable data were collected. By entering and finalizing the survey, participants agreed to participate. Those who did not finish the questionnaire were excluded from the study.

### Study procedure

The study was introduced by a researcher during an on-site meeting using a PowerPoint presentation. Participants who consented were asked to complete an online questionnaire via Google Forms on their smartphones. In the case of technological problems, the questionnaire was presented on paper. The survey included demographic questions (gender, region, professional role, and years of clinical work experience), followed by two diagnostic tests.

Each of the diagnostic tests consisted of a convenient sample of 21 high-quality still images of eardrums selected by two authors (MW and TL), to represent three diagnostic categories: normal eardrum, OME, and AOM, with seven images in each category (Figure S1). The same vignette was used for all images for both diagnostic tests: *‘William, 1.5-years old, comes to you at the health care centre. He has had earache and a cold for three days.’* During the test, the images were shown on a large screen in the presence of a researcher, and each image was displayed for 30 s. The participants delivered their answers in the online questionnaire or in the paper questionnaire.

#### Index test 1: without AI support

Each of the 21 eardrum images was shown to participants one at a time. For each image, one of three diagnostic categories (normal, OME, or AOM) was selected. They also rated their diagnostic confidence using a 6-point Likert scale, where 1 represented ‘not confident’ and 6 indicated ‘very confident’.

#### Index test 2: with AI support

Immediately following Index Test 1, the same set of 21 images was presented to participants in a new randomized order, now accompanied by fictional AI-generated diagnostic probability scores (Figure S2). Participants again recorded the diagnosis and confidence rating using the 6-point Likert scale. Importantly, participants were not informed in advance that the AI support was fictional or provided with details about its properties. The AI support were fictious to fully control its answers, which were made up by one of the authors (TL). For 18 of the 21 images, the AI support suggested the correct diagnosis, indicated through the highest probability score, while for 3 images, it indicated an incorrect diagnosis. This distribution simulated an overall AI diagnostic accuracy of 86%.

#### Reference standard

Reference diagnoses were established through consensus by a panel consisting of two general practitioners and one ear nose and throat specialist. The panel reviewed each image in combination with tympanometry results and clinical data. All images were obtained from a clinical study conducted in general practice, in which children 2–16 years presenting with otalgia were assessed using patient history, symptom evaluation, tympanometry (Entomed (Maico) Race Car Tympanometer), and digital still imaging with a straight fibre endoscope connected to a video camera (Hopkins, by Karl Storz, 1218AT 08 and Karl Storz Endovision, Telecam1 SL, 30 mm) [25].

### Statistical analysis

Demographic data, diagnoses, and confidence scores were compiled using *Microsoft Excel* and analysed using *SPSS* version 29.0.2.0 (20) and *Jamovi version* 2.3.28.0. As data were non-normally distributed, non-parametric tests were applied. Wilcoxon Signed-Rank Test was used to compare accuracy and confidence between test 1 and test 2. The Kruskal-Wallis test was used to identify differences between groups. A p-value < 0.05 was set as significant.

Participants were subdivided by gender and into four groups based on clinical work experience: (1) medical students, (2) physicians with ≤5 years of clinical work experience, (3) 6–10 years, and (4) ≥11 years. Since the ST group is diverse, containing both newly authorized physicians and physicians with many years of experience the use of work experience rather than work title (GP, ST, student) was chosen. Hence, most of the ST were found in group 2 and 3 (Table 1). Diagnostic accuracy with and without AI was compared across groups. Sensitivity, specificity, and predictive values were calculated using two classification schemes: (1) AOM versus normal and OME, as this distinction is crucial for deciding whether to prescribe antibiotics or not and (2) AOM and OME versus normal for potential application of AI support for screening purposes. *Jamovi* and the *MedDecide* module version 0.0.2 were used for these calculations.

**Table 1.** Demographics of participants.

	All participants (*N* = 259)	Clinical work experience % (N)			
Demographics	% (N)	Medical students (*N* = 73)	≤5 years (*N* = 78)	6–10 years (*N* = 74)	≥11 years (*N* = 34)
Women	64 (167)	71 (52)	59 (46)	66 (49)	59 (20)
Men	36 (92)	29 (21)	32 (41)	34 (25)	41 (14)
Medical students	30 (78)	99 (72)	8 (6)	0	0
ST	57 (148)	1 (1)	92 (72)	86 (64)	32 (11)
GP	13 (33)	0	0	14 (10)	68 (23)

Fleiss multi-rater kappa (κ) was used to assess inter-rater agreement using *Jamovi* with the *Seolmatrix* module version 3.7.2. Agreement levels were interpreted as: 0.00–0.20 none, 0.21–0.39 minimal, 0.40– 0.59 weak, 0.60–0.79 moderate, and 0.80–0.89 strong, and above 0.90 almost perfect [26].

To evaluate the influence of the AI tool on clinicians’ diagnostic decision-making, we applied a decision-pattern analysis described by Cabitza et al. based on the framework of Technological dominance [23]. The rationale for this analysis is that exposure to the diagnostic suggestion from the AI tool might lead the clinicians to change their original diagnoses. At times, this exposure will guide them to a correct diagnosis, but at the same time there is a risk it will mislead them. The decision table shows the clinicians’ original diagnoses, the AI tool’s suggestion, and the clinicians’ final decision after exposure to the AI support (Table 2). Consequently, there are eight possible patterns, depending on whether the diagnoses are correct or incorrect, which are illustrated in the decision table.

**Table 2.** Decision table.

Decision- pattern (number)	Human judgement (H)	AI support (AI)	Final decision (D)	Reliance pattern	Number of events (for all 21 images)
1	0	0	0	Detrimental reliance	411
2	0	0	1	Beneficial under-reliance	44
3	0	1	0	Detrimental self-reliance	746
4	0	1	1	Beneficial over-reliance	784
5	1	0	0	Detrimental over-reliance	174
6	1	0	1	Beneficial self-reliance	148
7	1	1	0	Detrimental under-reliance	284
8	1	1	1	Beneficial reliance	2848

Number of events for eight different combinations of diagnostic decisions. Correct (1) or incorrect (0) human judgement (H), AI support system (AI) and the final decision by human (D) when exposed to the AI support. Reliance pattern describes the eight combinations according to Cabitza et al.

Technological impact refers to the effect on diagnostic accuracy by the AI support system. It compares the probability of diagnostic error in Index Test 1 (without AI) to that in Index Test 2 (with AI). Values >1 imply that the positive aspects of dominance outweigh the negative ones. For a more detailed analysis of this dominance effect, automation bias and detrimental algorithmic aversion were also calculated. These metrics focus specifically on cases in which the clinicians’ diagnoses differed from the AI system’s suggestion (patterns 3–6).

*Automation bias* refers to the term for the negative impact of AI support, whereby clinicians make more errors when exposed to AI than when unaided. This measure includes all cases in which the clinician initially provided the correct diagnosis without AI, but the AI system suggested an incorrect one (patterns 5–6). Values greater than 1 indicate that the AI system misled clinicians toward incorrect diagnoses.

*Detrimental algorithmic aversion* describes the failure of the AI system to positively influence clinical decision-making. This metric includes cases where the clinician initially made an incorrect diagnosis without AI, but the AI system provided a correct suggestion (patterns 3–4). Values greater than 1 imply that clinicians disregarded the correct AI recommendation and maintained their incorrect decision.

## Results

### Demographics

A total of 259 participants were included in the study: 167 women and 92 men, representing three categories: 33 GPs, 148 STs in general practice and 78 medical students. Four subgroups were made according to work experience: medical students, ≤5 years, 6–10 years, and ≥11 years (Table 1).

### Diagnostic accuracy [database]

#### Index test 1: without AI support

The overall diagnostic accuracy across all participants was 64%. Accuracy was highest for AOM (77%), followed by normal eardrums (67%), and lowest for OME (46%) (Table 3). Overall inter-rater agreement was κ = 0.39. Agreement was highest for AOM (κ = 0.53), followed by normal eardrums (κ = 0.44), and lowest for OME (κ = 0.21).

**Table 3.** Diagnostic accuracy (%) and confidence (mean on a likert scale 1–6) overall and across the four groups of clinical work experience, without and with AI support and for all three diagnoses (AOM, OME, normal).

Diagnostic accuracy %	All *N* = 259	Medical students *N* = 73	≤5 years *N* = 78	6–10 years *N* = 74	≥11 years *N* = 34	p-value∞
Index Test 1	64	64	61	64	69	* *^1^*
Index Test 2	75	74	73	75	80	* *^2^*
p-value^	*	*	*	*	*	
AOM test 1	77	79	74	79	78	
AOM test 2	86	83	85	89	87	
p-value^	*	*	*	*	*	
OME test 1	46	48	41	48	54	* *^3^*
OME test 2	66	68	63	64	72	
p-value^	*	*	*	*	*	
Normal test 1	67	66	67	64	75	* *^4^*
Normal test 2	72	69	71	71	81	* *^5^*
p-value^	*	*	*	*		
Confidence Mean (median)						
Index Test 1	3.9 (4)	4.0 (4)	3.7 (4)	3.9 (4)	4.2 (4)	* *^6^*
Index Test 2	4.2 (4)	4.3 (4)	3.9 (5)	4.2 (4)	4.5 (4)	* *^7^*
p-value^	*	*	*	*	*	
AOM test 1	4.3 (5)	4.5 (5)	4.0 (5)	4.3 (5)	4.5 (4,5)	* *^8^*
AOM test 2	4.5 (5)	4.6 (5)	4.2 (5)	4.6 (5)	4.7 (5)	* *^9^*
p-value^	*	*	*	*	*	
OME test 1	3.5 (3)	3.5 (3)	3.3 (3,5)	3.5 (4)	3.8 (4)	
OME test 2	3.8 (4)	4.1 (4)	3.6 (4)	3.8 (4)	4.1 (4)	* *^10^*
p-value^	*	*	*	*		
Normal test 1	3.9 (4)	3.8 (4)	3.7 (4)	3.9 (4)	4.2 (4)	* *^11^*
Normal test 2	4.2 (5)	4.2 (5)	4.0 (5)	4.3 (4)	4.6 (4,5)	* *^12^*
p-value^	*	*	*	*	*	

* <0.05.

^^^ Difference of accuracy when AI is used using Wilcoxon Signed Rank Test.

^∞^ Difference within all groups using Kruskal-Wallis.

Difference between separate groups in Accuracy were significant in the following cases.

1. group 2 and 4.

2. group 1 and 4, 2 and 4, 3 and 4.

3. group 1 and 2, 2 and 3, 2 and 4.

4. group 3 and 4.

5. group 1 and 4, 3 and 4.

Difference between separate groups in Confidence were significant in the following cases.

6. group 2 and 4.

7. group 2 and 4.

8. group 2 and 4, 1 and 2.

9. group 2 and 3, 2 and 4.

10. group 1 and 2, 2 and 4.

11. group 2 and 4.

12. group 2 and 4.

Among the groups based on clinical experience, the highest accuracy was found for participants with ≥11 years of clinical experience (69%), while those with ≤5 years had the lowest (61%) (Table 3). No significant difference in accuracy was found between women and men.

For all participants, sensitivity for detecting AOM was 77% and specificity 82% (Table 4). Positive predictive value (PPV) and negative predictive value (NPV) were 68% and 88%, respectively.

**Table 4.** Sensitivity, specificity and predictive values without and with AI support for screening out AOM from OME and normal and for and for screening out normal from AOM and OME. Values for the AI support system are presented in the third column.

AOM vs OME and Normal	Index Test 1 without AI	Index Test 2 with AI	AI support
Sensitivity (%)	77	86	86
Specificity (%)	82	84	86
Positive predictive value (%)	68	73	75
Negative predictive value (%)	88	92	92
AOM and OME vs Normal	Index Test 1 without AI	Index Test 2 with AI	AI support
Sensitivity (%)	88	97	86
Specificity (%)	67	72	86
Positive predictive value (%)	84	87	75
Negative predictive value (%)	74	92	92

When AOM and OME were combined as ‘abnormal’, sensitivity and PPV increased, while specificity and negative predictive values (NPV) decreased (Table 4).

#### Index test 2: with AI support

Overall diagnostic accuracy improved significantly with AI support, increasing from 64% to 75% *(p* < 0.05). Significant improvements were observed across all clinical work experience groups, genders, and diagnostic categories (Table 3). Participants with ≥11 years of clinical experience maintained the highest overall accuracy, and women significantly outperformed men significantly (77% vs. 73%, *p* < 0.05).

Inter-rater agreement also improved with AI support, increasing to κ = 0.60 overall. Agreement for AOM increased to κ = 0.64, for normal eardrums to κ = 0.72 and for OME to κ = 0.46.

For AOM, overall sensitivity increased from 77% to 86%, and specificity from 82% to 84% (Table 4). When AOM and OME were grouped as ‘abnormal’, both sensitivity and PPV increased, while specificity and NPV decreased.

Diagnostic accuracy for the 18 images associated with correct fictional AI diagnoses was significantly higher than for the three images associated with incorrect AI diagnoses. This pattern was observed both overall and across all clinical experience groups in Index Test 1 (without AI support). In Index Test 2 (with AI support), the difference became even more pronounced (Table 5).

**Table 5.** Mean accuracy and confidence when AI are correct (18 images) versus incorrect (3 images) for overall and across the 4 groups of clinical work experience.

Accuracy, mean	Overall	Medical students	≤5 years	6–10 years	≥11 years
N	259	73	78	74	34
When AI is correct*					
Index Test 1	67%	69%	64%	67%	71%
Index Test 2	83%	83%	81%	83%	87%
When AI is Incorrect*					
Index Test 1	41%	35%	40%	42%	56%
Index Test 2	25%	17%	24%	27%	36%
Confidence, mean					
When AI is correct*					
Index Test 1	4.0	3.8	4.0	4.1	4.1
Index Test 2	4.3	4.2	4.4	4.4	4.4
When AI is incorrect					
Index Test 1	3.2	2.9	3.3	3.3	3.3
Index Test 2	3.2	2.9	3.3	3.4	3.2

Difference of Accuracy and Confidence between without and with AI support is significant for all except for Confidence when AI is incorrect. *p < 0.05. Index test 1 = without AI, index test 2 = with AI.

### Diagnostic confidence

#### Index test 1: without AI support

The mean diagnostic confidence rating across all participants and images was 3.9 on a 6-point Likert scale. Confidence was highest for AOM (mean 4.3), lowest for OME (mean 3.5), and intermediate for normal eardrums (mean 3.9). Participants with ≥11 years of clinical experience reported the highest confidence levels overall and across all diagnostic categories, while those with ≤5 years of experience reported the lowest (Table 3). Women rated their diagnostic confidence lower than men (3.8 vs. 4.1, *p* < 0.05).

#### Index test 2: with AI support

Diagnostic confidence improved significantly across all groups and all diagnoses, increasing from 3.9 to 4.2. Those with ≥11 years of clinical experience maintained the highest confidence, and those with ≤5 years the lowest (Table 3). Although women continued to report lower, but not significantly lower, confidence than men (*p* = 0.05), they achieved slightly higher diagnostic accuracy than men in Index Test 2 (*p* < 0.05).

### Influence of AI support on participants

#### Technology dominance

The analysis showed a *technological impact* well above 1 across all clinical work experience groups (Table 6). *Automation bias* was >1 for all groups except those with ≥11 years of experience, with the highest value observed among medical students, decreasing gradually with increasing clinical experience.

**Table 6.** Technological impact, automation bias and detrimental algorithmic aversion overall, for the 4 groups of clinical work experience and for men and women.

	All	Medical students	≤5 years	6–10 years	≥11 years	Women	Men
Technological impact	1.4	1.2	1.4	1.4	1.4	1.4	1.3
Automation bias	1.2	1.4	1.2	1.1	0.9	1.2	1.2
Detrimental algorithmic aversion	0.94	1.1	1.0	0.75	0.68	0.82	1.2

Technological impact: Values >1 imply that the positive aspects of dominance outweigh the negative ones.

Automation bias: Values >1 imply that AI is misleading the clinician towards the wrong diagnosis.

Detrimental algorithmic aversion: Values >1 imply that the AI tool fails in exerting positive dominance, instead clinicians hold onto their incorrect decisions.

*Detrimental algorithmic aversion* was also greater than 1 for medical students and showed a similar decline with clinical experience increased. A gender difference was observed, where women had an *algorithmic aversion* value below 1, whereas men had a value above 1.

## Discussion

This study investigated the diagnostic accuracy for common types of OM in general practice and among medical students. Using high-quality eardrum images without mobility information, participants’ ability to assess eardrum appearance was evaluated, revealing a modest overall accuracy of 63%. Diagnostic accuracy was significantly improved when fictional AI support was provided, and a generally positive influence was noted across all participant groups.

The highest accuracy in both assessments was achieved by the most experienced physicians. Despite their expertise, they still benefited significantly from AI support and were not misled by incorrect AI suggestions. The least experienced physicians, who initially exhibited the lowest accuracy, demonstrated the greatest improvement when AI support was introduced. Correct AI recommendations were associated with improved accuracy, while incorrect ones led to a decrease. A modest but significant increase in diagnostic confidence was also recorded with AI support.

### Diagnostic accuracy [database]

We show that the overall diagnostic accuracy is negatively impacted by low accuracy in detecting OME and normal eardrums. It is known that combining otoscopy with tympanometry or pneumatic otoscopy significantly improves the evaluation of OME, with a sensitivity of 94% and a specificity of 80% for pneumatic otoscopy [27]. In our study, information about ear drum mobility was not available but high-quality digital images were used instead. Digital otoscopy has shown promising results in improving diagnostic accuracy [14,28]. The GP in our study detected OME at a rate comparable to those in the study by Buchanan et al. where only oto-endoscopic photographs were assessed [10]. In comparison, a clinical study where GP used pneumatic otoscopy showed an overall accuracy of 67% which is comparable to our results [29]. In a study by Blomgren et al. experienced GPs achieved about 80% specificity in diagnosing AOM using pneumatic otoscopy and tympanometry [5]. Our specificity in diagnosing AOM was 82% (84% with AI support), supporting the notion of high-quality eardrum images as a positive influence in the classification of OM.

Over-diagnosis is a known problem related to diagnostic uncertainty in OM diagnostics [30]. Our findings of a low PPV for diagnosing AOM imply that inaccurate diagnosis led to over-diagnosis. Over-diagnosis, i.e. diagnosing OME as AOM, is likely the reason for the improved PPV scores when we group AOM and OME together as ‘abnormal’ to screen out normal ears.

Overall inter-rater agreement was minimal without AI support but increased to moderate with AI support. A low inter-rater agreement can either indicate that the participants’ knowledge about important features when assessing OM was low or that diagnostic criteria used were dispersed. In Sweden a guideline for treatment of AOM was published in 2010 including diagnostic criteria [31]. We therefore hypothesise that knowledge about how to diagnose OM correctly is insufficient among physicians. Blomgren et al. demonstrated a significant reduction in AOM diagnosis when correct diagnostic criteria and appropriate equipment were utilized [4]. Clinicians have previously been found to give redness a high diagnostic value when diagnosing AOM. The most critical feature for diagnosing AOM is, however, the presence of an opaque, bulging eardrum with a cloudy (non-transparent) appearance [32]. The specific features the participants in our study relied on when assessing the eardrum were not investigated in our study.

Hypothetically, if a high inter-rater agreement had been the case in our results, together with the modest accuracy, it would suggest that participants agreed on diagnostic criteria that did not align with our reference diagnosis. Our findings of low agreement indicate a need for enhanced education in OM diagnostics during medical school and for ST. We show improved inter-rater agreement when participants employed the AI support system.

### Diagnostic confidence

The clinicians’ confidence is an important factor in clinical decision-making. When counselling patients, a physician’s confidence in their diagnosis can enhance patient trust and adherence to treatment recommendations. However, excessive confidence may hinder physicians from recognising their own mistakes and correcting diagnostic errors [33,34]. We analysed the participants’ confidence in making diagnosis and found lower confidence in classifying OME and normal eardrums compared to AOM. Diagnostic accuracy was higher for AOM, and in that sense, the participants’ confidence appears to reflect their ability. The most experienced group demonstrated both the highest diagnostic accuracy and the highest confidence.

Diagnostic confidence and performance in our study do not appear to interact in the same way for women and men. We show, in line with other studies, that men rated higher diagnostic confidence than women both without and with AI support, while female physicians had a slightly greater improvement in diagnostic performance with AI support than male physicians [35–37].

Improving confidence in OM diagnostics can be achieved through training, as shown in studies by You et al. [38]. The use of digital otoscopy can also improve confidence [39]. In our study, we observed a small improvement in confidence when participants were aided by an AI support, particularly for OME and normal eardrums. The most experienced physicians showed the highest confidence, indicating the need for training among ST.

### Influence of AI support on participants

The AI support in our study, with an accuracy of 86%, improved both diagnostic confidence and accuracy, even though the participants had no information about the AI support before they were exposed to it. With the aid of AI support, the most experienced physicians achieved the highest accuracy and demonstrated the most beneficial profile of technological influence. The results align with previous knowledge about factors influencing over-reliance on AI decision aids, where task experience plays a central role [23]. Still, the estimated *technological impact* was equally high for them and for the least experienced physicians with low confidence and accuracy. The medical students, followed by the least experienced physicians had the highest *automation bias* and *detrimental algorithmic aversion*, suggesting that they tended to follow AI support more often when it was wrong, and failing to change their diagnosis when it was right. The least experienced physicians consisted of individuals in their early ST in general practice, still with limited clinical experience. The findings support the previously discussed need for enhanced education and recurring training.

### Strengths and weaknesses

Given the limited number of studies performed assessing diagnostic accuracy for OM in general practice, our study provides important insights. A strength of this study is the use of high-quality images, enabling core analysis of the skills in assessing eardrum appearance. We also used a high-standard reference diagnosis. In a clinical setting, other aspects of diagnostic challenges would likely affect the results, such as earwax, an irritable child, or poor lighting.

Our study has weaknesses. First, we only used still images. A video showing pneumatic otoscopy or the addition of tympanometry results would likely improve accuracy, especially for normal eardrums and OME.

Secondly, our study was conducted on a limited population of physicians. Selection bias may have influenced the results of medical students since they were invited to participate. The ST and GP were present at regular educational meetings where the risk of selection bias is lower.

In the cases of incorrect AI classification, these images seemed to be more challenging to diagnose by the participants (low accuracy in Index test 1), making it more difficult for the participants to detect when the AI support suggestions were incorrect. This could lead to an excessive increase in automation bias. Of these three images (Figure S3), one shows a translucent eardrum in a normal position without any signs of middle ear effusion. One is a severely deformed, chagrinated eardrum with swollen keratin patches and no distinguishable handle of malleus, obviously bulging. The third image is an opaque and cloudy eardrum in a normal, or rather retracted position, handle of malleus easily recognizable. According to current diagnostic guidelines these images would be diagnosed as normal, AOM and OME.

We grouped participants based on years of clinical experience, which does not necessarily correlate with experience in assessing OM. Even though the definition of clinical experience was not clearly defined, the tendencies in our results with improved accuracy and confidence correlating positively with work-life experience, are likely the result of training. As shown in Table 1, one of the ST physicians mistakenly categorized themselves as a medical student when registering clinical experience in the questionnaire. Although this may have affected the results, we believe the impact is not significant.

The visual quality of the PowerPoint presentation varied since we did not use standardised technique for presentation, and there was a noticeable difference in quality between projectors. However, no significant differences in accuracy were observed across sessions (analyses not presented), leading us to assume that visual quality did not significantly affect performance.

Images were presented twice, potentially improving accuracy in Index Test 2 due to familiarity with previously seen images and the impact of repeated exposure [40]. At the same time, accuracy may decrease as the participants might lose concentration over time [41]. However, no accuracy change was observed over time, indicating that these concerns were negligible.

### Clinical impact and future research

Our study provides valuable insights into the diagnostic accuracy for OM in primary care and among medical students. We demonstrate the need to improve diagnostic accuracy of OM and reduce over-diagnosis in general practice. Introducing digital imaging together with AI could help facilitate decision-making, and we observed significant improvements using this approach. Education and training are other measures that have been discussed for the past 20–30 years, with several studies showing the positive effect of training [42,43]. Digital training solutions for continuous education could be easy to use and time efficient. Furthermore, AI has the potential to act as a teacher in the diagnosis of OM if used in clinical care.

Future research should explore several key questions regarding the integration of AI in medical practice. One area of interest is whether AI should be considered an expert that can replace the physician or whether be used as a support system. This could be especially important for rural areas with limited access to physicians. Additionally, studies should investigate whether enhancing teaching methods using digital videos or still images is more effective than using AI. The potential for AI support in medical education also warrants examination. Furthermore, understanding how AI influences physicians’ decision-making processes in clinical environments is crucial. What impact could an AI system with additional explanation of the selected diagnosis (explainable AI) have on the user’s diagnostic accuracy and confidence?

## Conclusion

A modest level of diagnostic accuracy for OM was demonstrated among primary care physicians and medical students, consistent with findings from previous studies. Accuracy, diagnostic confidence, and inter-rater agreement all improved with the use of a fictitious AI support, and a reduction in over-diagnosis was observed. The highest performance was achieved by the most experienced physicians. In contrast, less experienced participants were more susceptible to being misled by incorrect AI suggestions.

## Supplementary Material

Supplemental Material

## Data Availability

Data are available on request from the corresponding author.
