# Supplementary material for: Diagnostic accuracy of otitis media with and without a fictitious AI support among physicians in primary care and medical students
Source: Scand J Prim Health Care. 2025 Oct 15;44(1):1–13. doi: 10.1080/02813432.2025.2571936 (PMC12918355; doi:10.1080/02813432.2025.2571936)
Supplement: Supplemental Material [file IPRI_A_2571936_SM2279.docx]

# Supplementary material


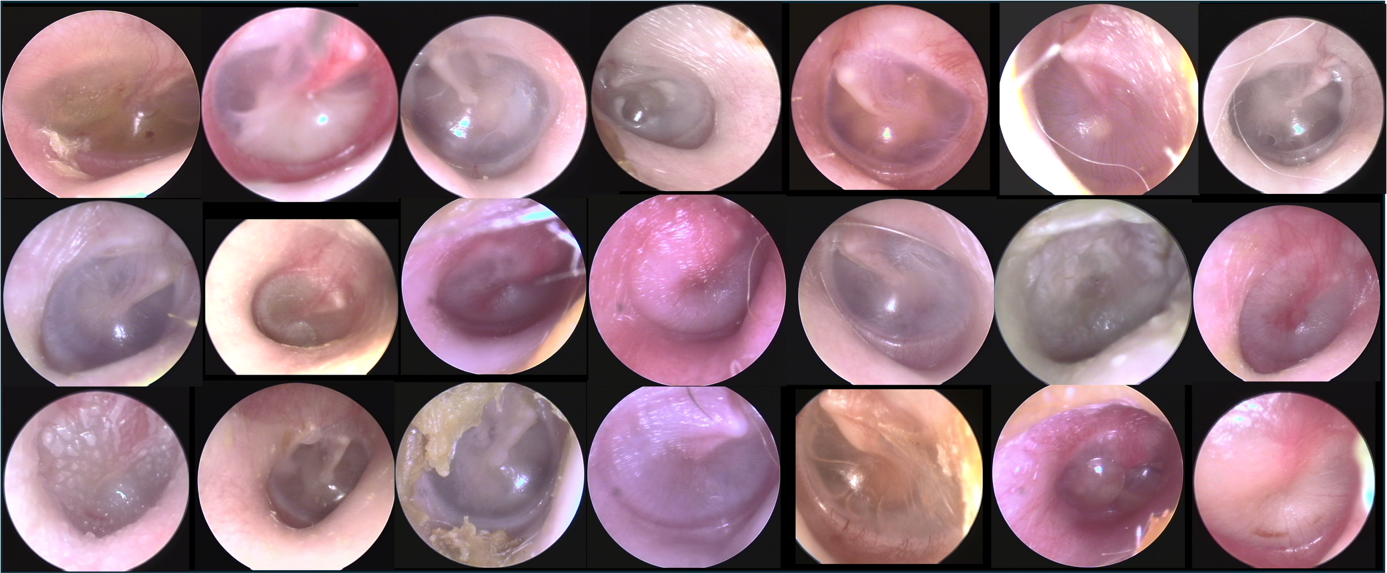


Figure S1. All 21 tympanic membrane images.

*
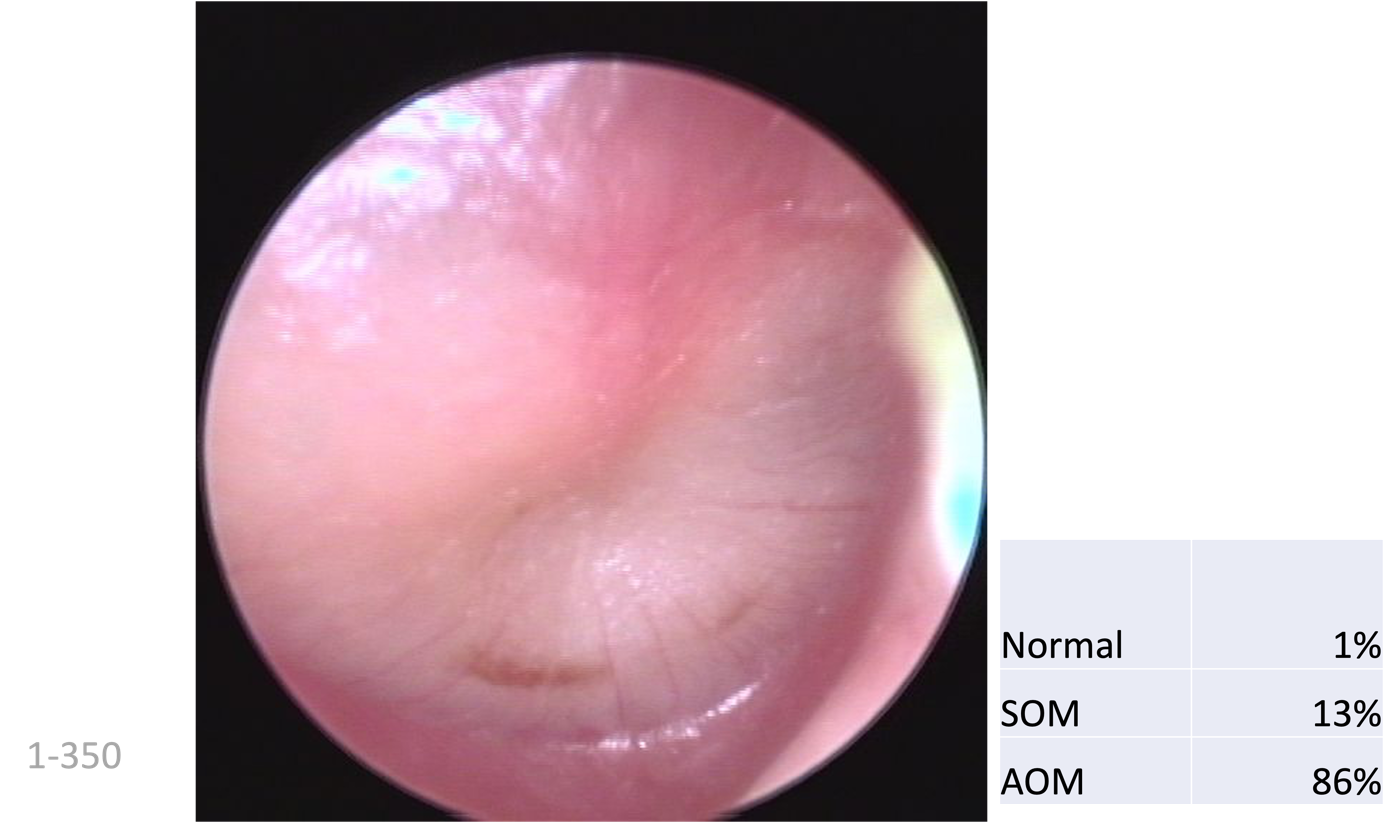
*

*Figure S2. Image of how the suggestion from the AI support was presented to the participants. SOM is Swedish terminology for OME.*

*Figure S3. The three images where the AI support suggested an incorrect diagnosis. SOM is Swedish terminology for OME.*
